# Characterizing the Elemental Metabolome in *Asparagus officinalis* with Multielemental Speciation Analysis

**DOI:** 10.1021/acs.jafc.6c10753

**Published:** 2026-08-28

**Authors:** Ivan Milovanovic, Lorenz Steiner, Claudia Kerschbaumer, Walter Goessler, Bassam Lajin

**Affiliations:** † Innovation Centre of the Faculty of Technology and Metallurgy, 54801 University of Belgrade, Karnegijeva 4, 11000 Belgrade, Serbia; ‡ Institute of Chemistry, Analytical Chemistry for the Health and Environment, 27267 University of Graz, Universitaetsplatz 1, 8010 Graz, Austria; § Institute of Chemistry, ChromICP, University of Graz, Universitaetsplatz 1, 8010 Graz, Austria

**Keywords:** sulfur speciation, ICP–MS/MS, HPLC, arsenic speciation, food metabolomics

## Abstract

We report a multielemental and quantitative speciation analysis study with chromatographic inductively coupled plasma–mass spectrometry (ICP–MS/MS) detection involving four elements (sulfur, selenium, arsenic, and phosphorus) in *Asparagus officinalis*. The samples were locally purchased and labeled to originate from different regions in two continents. Major compounds were identified and quantified with confirmation based on chemical synthesis in pure form. Nontargeted analysis revealed significant quantitative and qualitative variation for the elemental metabolomes among samples, particularly sulfur and arsenic. Sharp contrast was observed in the diversity of the profiles between sulfur and selenium, providing insight into metabolism selectivity in *Asparagus*. Inorganic and methylated arsenic were both detected, but their ratios showed wide variation between samples. The present work shows the potential of multielemental speciation analysis with ICP–MS/MS as a novel dimension for food characterization based on studying heteroatom-tagged metabolome.

## Graphical abstract

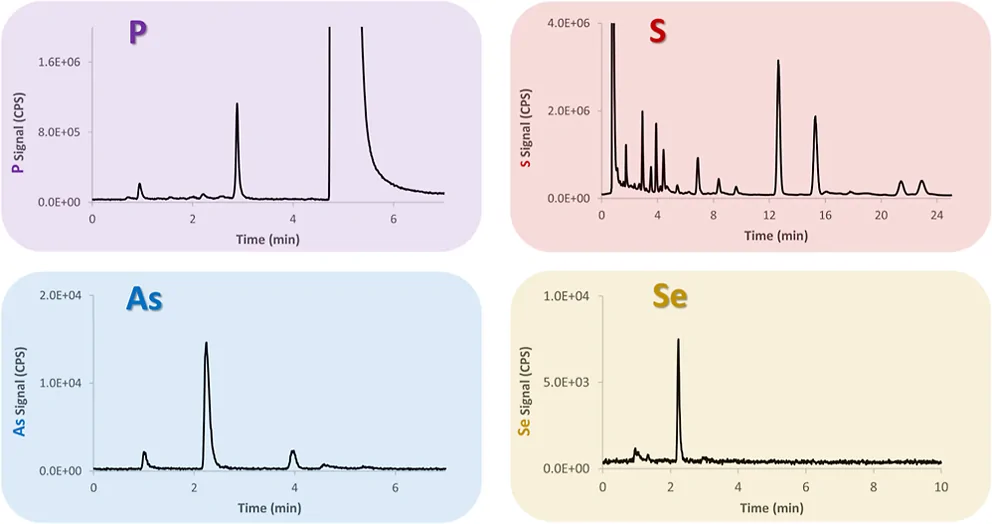

## 1 Introduction

Metabolomic analysis of food enables effective monitoring of food quality and safety, the discovery of novel bioactive components that advance nutritional knowledge, and comprehensive assessment of the overall load of environmental pollutants.^1,2^ Coupling chromatographic separation with element-selective detection enables a unique approach to studying the metabolome in food, allowing selective detection of novel and potentially bioactive compounds tagged with a heteroatom. Inductively coupled plasma–mass spectrometry (ICP–MS) is the most commonly coupled element-selective detector with liquid chromatography, as it offers multielemental monitoring with limits of detection <1.0 μg·L^–1^, quantitative analysis with higher resistance to matrix effect than conventional molecular mass spectrometric techniques used in metabolomic analysis such as electrospray-ionization mass spectrometry (ESI–MS), and molecule-independent signal response.^3^

The overwhelming majority of applications of HPLC-ICP–MS have been restricted to compounds tagged with arsenic or selenium because of major polyatomic interferences. However, the relatively recent introduction of tandem mass spectrometry to the technique (ICP–MS/MS) enabled extension of applicability to nonmetal elements such as sulfur, which is a particularly relevant element of interest in asparagus.

The number of applications of HPLC-ICP–MS/MS in food analysis has been relatively limited. In particular, we found very few studies exploring asparagus using HPLC-ICP–MS, mostly involving arsenic rather than major elements such as sulfur or other nonmetals, as a single quadrupole ICP–MS was used.^4^ In a proof-of-concept application, we previously showed the potential of ICP–MS/MS in multielemental speciation analysis in mushrooms, revealing the great diversity in elemental profiles among a variety of edible and inedible mushroom species.^5^ Herein, we apply a multielemental speciation analysis approach on cultivated commercially available whole spears of green asparagus (*Asparagus officinalis*) locally purchased and labeled to originate from different regions across Europe and South America. The aims of the present study were (1) to acquire novel elemental profiles for four elements of interest, namely, phosphorus, arsenic, selenium, and sulfur, which were not previously investigated with HPLC-ICP–MS in asparagus; (2) to compare the metabolomic diversity between pairs of chemically related elements (e.g., sulfur and selenium); and (3) to demonstrate the general applicability of HPLC-ICP–MS/MS for studying variability in the metabolomic profiles of bioactive components in food.

## 2 Materials and Methods

Green asparagus samples (*Asparagus officinalis*) labeled to originate from seven regions across Europe and South America (Mexico, Peru, Greece, Macedonia, Italy, Serbia, and Slovenia) were locally purchased, anonymously coded as R1-R7, and subjected to freeze-drying and homogenization. Extraction was performed using pure water (18.2 MΩ·cm) by suspending 0.5 g of lyophilized and ground sample in 10 mL in a 15 mL polypropylene tube and sonication for 30 min, followed by shaking for 24 h in a cross-shaped rotating device. Ascorbic acid was incorporated as an antioxidant at 2% w/v concentration in the extraction procedure in selected experiments in order to ensure repeatability of element speciation profiles.

Determination of total element concentration for 30 elements in portions of the lyophilized samples (250 mg) was performed using an ICP–MS system (Agilent 7900 ICPMS, Agilent Technologies, Waldbronn, Germany) following microwave-assisted digestion with 5 mL nitric acid 65% w/w (Carl Roth, Karlsruhe, Germany) using an Ultraclave IV digestion system (MLS GmbH, Leutkirch, Germany) at 250 °C for 20 min under 40 bar argon gas. Digested samples were diluted to 50 mL with pure water and analyzed in the no-gas and/or helium mode (3.5 mL·min^–1^) while employing germanium and indium as internal standards. Determination was performed in triplicate to examine repeatability, and standard reference material (NIST 1643a) and drift standards were included for quality control.

For speciation analysis, extracts, prepared as mentioned above, were centrifuged at 5000 × g, and 0.5 mL was transferred to 0.7 mL polypropylene HPLC vials (Bruckner, Linz, Austria) for analysis. Chromatographic separation was performed on an Agilent 1100 system (Agilent Technologies, Waldbronn, Germany). A reversed-phase column (YMC Triart-C18, 150 × 2.1 mm, 3 μm particle size) was employed for all experiments, with isocratic application of various mobile phases to ensure retention of positively charged, negatively charged, and neutral compounds, namely, 0.4% heptafluorobutyric acid (pH 1.5), 0.2% tripropylamine (pH 9.5, adjusted with acetic acid), and 0.1% formic acid (pH 2.7). The column was held at a temperature of 50 °C, the mobile phase flow rate was 0.4 mL·min^–1^, and the injection volume was 1.0 μL. To achieve minimal limits of detection for speciation analysis, an inductively coupled plasma tandem mass spectrometry (ICP–MS/MS) system was used as the chromatographic detector (Agilent 8900 ICPQQQ, Agilent Technologies, Waldbronn, Germany) operated in the oxygen mode (0.3 mL·min^–1^) to produce mass shifts, *m*/*z* 32 → 48 for sulfur, 31 → 47 for phosphorus, 80 → 96 for selenium, and 75 → 91 for arsenic. The main instrumental parameters were as follows: RF power: 1550 W; RF matching: 1.7; sampling depth: 5.0 mm; nebulizer gas flow rate: 0.65 L·min^‑1^ and makeup gas flow rate: 0.15 L·min^‑1^; and optional gas flow (CO_2_): 0.20 L·min^–1^. The limit of detection (dry mass basis) for As was 1.0 μg·As·kg^‑1^ (0.05 μg·As·L^–1^ in water extract, based on S/N 3 definition, i.v. 2 μL), for Se 6.0 μg·Se·kg^1–^ (0.3 μg·Se·L^–1^, i.v. 5 μL), for P 2.0 mg·P·kg^1–^ (0.1 mg·P·L^–1^, i.v. 1 μL), and for S 1.0 mg·S·kg^1–^ (0.05 mg·S·L^–1^, i.v. 1 μL).

Peak assignment was performed by coinjection experiments using pure standards commercially available (Sigma-Aldrich, Steinheim, Germany) or synthesized in-house according to previously described procedures.^6^ Additionally, molecular tandem mass spectrometry was employed to confirm identification (Ultivo LC/TQ system (Agilent Technologies, Waldbronn, Germany)). For molecular mass spectrometric identification of sulfur compounds, multiple mass transitions were employed (retrieved from the human metabolome database^7^), including those corresponding to the ^34^S isotope.

All concentrations in the present work were reported as element mass per kilogram (dry mass basis).

## 3 Results and Discussion

Total element determination for 29 elements showed little variation among the samples in the concentrations of the majority of elements, especially alkaline, alkaline earth, and nonmetals such as sulfur and phosphorus (Table 1), whereas trace element concentrations were in line with previously reported literature values (summarized in ref 8). Selenium concentrations were within a relatively narrow range (0.1–0.4 mg·kg^1–^, dry mass basis). On the other hand, arsenic concentrations were generally low but varied within a relatively wide concentration range 11–152 μg·kg^1–^.

**Table 1:** Total Element Content of 29 Elements in Commercially Available Asparagus Labeled to Originate from Various Regions across Europe and South America^c^

	R1^a^	R2	R3	R4	R5	R6	R7	NIST 1643f^b^
g kg^1–^								mg kg^1–^
Na	0.64	0.11	0.64	0.24	0.50	0.36	0.74	19 (19)
Mg	1.8	1.1	2.1	1.4	1.8	2.0	1.9	7.7 (7.4)
P	5.2	5.4	6.3	6.0	5.3	5.3	5.9	58
S	5.7	5.5	5.5	5.7	4.8	4.4	6.3	2.5
K	29	27	35	32	30	27	30	1.8 (1.9)
Ca	2.5	3.2	2.3	3.8	2.2	2.5	3.1	32 (29)
mg kg^1–^								μg·kg^1–^
Al	32	21	63	62	225	62	57	185 (134)
B	24	19	32	18	18	27	26	163 (152)
Cr	0.17	0.49	0.31	0.18	0.62	0.13	0.42	23 (18)
V	0.12	0.03	0.24	0.14	0.33	0.19	0.11	37 (36)
Mn	27	47	27	23	25	40	14	43 (37)
Fe	48	51	78	91	168	74	77	120 (93)
Co	0.49	0.09	0.21	0.06	0.09	0.28	0.10	27 (25)
Cu	12	14	14	19	16	12	13	26 (22)
Zn	54	58	54	70	51	51	62	104 (74)
Se	0.27	0.38	0.25	0.09	0.11	0.39	0.35	12 (12)
Rb	6.6	8.7	7.3	23	1.6	7.0	7.1	17 (13)
Sr	12	13	6.1	13	5.4	7.5	12	310 (314)
Mo	0.52	0.19	0.85	0.17	0.55	0.27	0.84	114 (115)
Ba	1.3	2.5	1.1	2.0	3.4	1.2	1.2	522 (518)
Li	0.11	0.22	0.36	0.03	0.27	0.12	0.23	16 (17)
μg kg^1–^								
As	18	11	152	14	48	13	34	62 (57)
Sb	8.1	18	34	4.1	5.0	5.7	10	53 (55)
Pb	36	52	106	54	109	17	30	18 (18)
U	7.0	2.0	3.6	2.7	7.1	2.6	2.7	0.01
Tl	0.8	1.3	1.9	9.3	2.1	1.3	3.0	6.4 (6.9)
Gd	2.4	1.3	3.2	6.0	1.9	3.4	2.7	0.01
Ag	4.8	5.4	1.7	4.6	3.3	5.1	3.5	1.0 (0.97)
Sn	9.2	3.4	7.8	9.0	14	59	3.9	0.2

a Each sample was digested and measured in triplicate to assess repeatability (RSD generally <10% across all elements).

b Available certified concentrations for the NIST 1643f standard reference material are provided in brackets.

c Concentrations are reported as dry mass basis.

Asparagus samples displayed consistent patterns in terms of the diversity in the elemental profiles. The largest diversity among the studied elements was observed for sulfur (Figure 1) with >10 compounds detected. However, the sum of six major compounds was found to dominate 40%–60% of total extractable sulfur. Based on MS/MS as well as coelution experiments, these compounds were identified to be inorganic sulfate, asparagusic acid, two steric isomers of asparagusic acid *S*-oxide, and methionine (Supplementary Figure S1).

**Figure 1:**
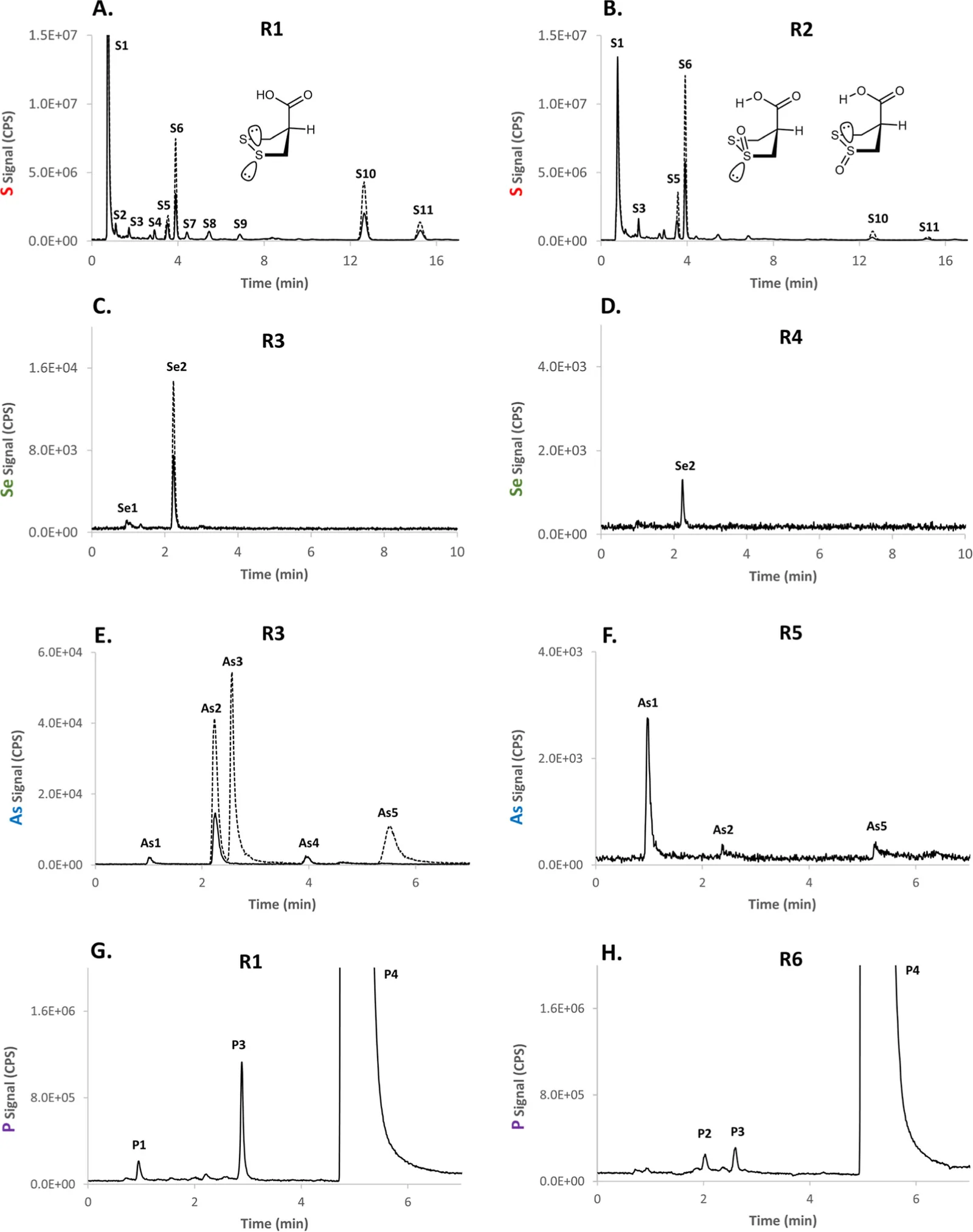
Multielement speciation in *A. officinalis* samples (R1-R6), see text, showing similarity and contrast in sulfur (A,B), selenium (C,D), arsenic (E,F), and phosphorus (G,H) profiles. Compounds were identified based on spiking experiments (shown as dashed lines) and molecular tandem mass spectrometry (see Supplementary Figure S1). Identified species were abbreviated as follows: S1, sulfate; S2, dimethyl sulfoxide; S3, methionine sulfoxide; S5/S6, diastereomers of asparagusic acid S-oxide; S10, asparagusic acid; S11, methionine; Se2, selenomethionine; As1, arsenite (AsIII); As2, dimethylarsinic acid (DMA); As3, methylarsonic acid; As4, unknown arsenic compound; As5, arsenate (As­(V)); and P4, phosphate. Chromatographic conditions: stationary phase YMC C18 triart 150 mm × 2.1 mm, 3 μm, mobile phase: (0.4% v/v heptafluorobutyric acid pH 1.5 (A,B); 0.1% formic acid pH 2.2 (C,D); 0.2% tripropylamine-acetate pH 9.5 (E,F,G,H). The structures of asparagusic acid and its oxide diastereomers are shown in A and B, respectively.

Table 2 shows quantitative information for all identified sulfur species as well as total sulfur concentrations. Sulfur metabolomic profiles are provided for all samples in Supplementary Figure S2. The identification of asparagusic acid and the steric isomers of its oxide form has been previously reported.^9^

**Table 2:** Concentrations (in g·S·kg^–1^ Dry Mass Basis) of Sulfur Compounds in *A. officinalis*

	T-S	E-S^b^	sulfate	AA	AAO	Met	MetO	DMSO	sum
R1^a^	5.7	3.7 (64)	1.3 (36)	0.28 (7.6)	0.25 (6.7)	0.11 (3.1)	0.03 (0.8)	0.03 (0.9)	3.2 (88)
R2	5.5	2.6 (47)	0.6 (22)	0.02 (1.0)	0.36 (14)	0.01 (0.5)	0.03 (1.0)	0.05 (1.8)	2.2 (86)
R3	5.5	3.2 (58)	1.4 (45)	0.26 (8.2)	0.02 (0.7)	0.30 (9.2)	0.04 (1.2)	0.02 (0.5)	3.0 (94)
R4	5.7	4.0 (69)	1.2 (30)	0.51 (13)	0.07 (1.7)	0.30 (7.5)	0.05 (1.3)	0.02 (0.6)	3.4 (86)
R5	4.8	3.9 (81)	0.4 (11)	1.3 (35)	0.04 (1.1)	0.33 (8.4)	0.03 (0.7)	0.01 (0.2)	3.3 (85)
R6	4.4	3.2 (74)	0.4 (14)	0.99 (31)	0.13 (3.9)	0.29 (9.0)	0.03 (1.0)	0.01 (0.3)	2.7 (85)
R7	6.1	4.2 (69)	1.7 (42)	0.41 (10)	0.11 (2.6)	0.28 (6.6)	0.08 (1.9)	0.03 (0.8)	3.8 (92)

a Abbreviations: R1–R7 indicate commercially available asparagus labeled to originate from various regions (see text); T-S, total sulfur; E-S, total water extractable sulfur; AA, asparagusic acid; AAO, asparagusic acid S-oxide (sum of the two diastereomers); Met, methionine; MetO, methionine sulfoxide; DMSO, dimethylsulfoxide; and Sum, sum of all sulfur species detected including unidentified species.

b Numbers in parentheses show percentages of extractable sulfur. For E-S, the percentage of total sulfur was calculated. Repeatability was checked by performing multiple extractions (*n* = 3) with RSD % generally <15%.

Interestingly, the significant diversity in the sulfur metabolome in asparagus was found to be in sharp contrast with that in the selenium metabolome, where a single selenium compound, identified to be selenomethionine, was the sole major selenium compound detected in all samples (Figure 1). The discrepancy in diversity between selenium and sulfur metabolomes is somewhat surprising since selenium and sulfur share chemical properties, and many examples of selenium analogues for sulfur compounds are known in nature.^10^ The primary example of this is the amino acid methionine and its selenium analogue selenomethionine, which are thought to be similarly incorporated into proteins.^11^ Indeed, the contribution of selenomethionine to total selenium in asparagus was found to significantly increase from less than 20% up to 70% following extraction in the presence of proteinase K, indicating that the majority of selenium in asparagus is protein-bound in the form of selenomethionine (Table 3). The observed low diversity in the selenium metabolome compared with that of sulfur suggests that the diverse sulfur metabolic pathways in asparagus, particularly the biosynthesis of the major compound asparagusic acid, might be selective to sulfur over selenium. However, it must be emphasized that for a more thorough examination of this interesting aspect, the selenium profile must be investigated following artificial supplementation of asparagus with high selenium concentrations and then compared with the sulfur profile using HPLC-ICP–MS/MS in order to definitively conclude that the lack of selenium metabolites is due to selectivity for sulfur over selenium rather than merely due to limited selenium availability in the cultivation environment, and this warrants future investigation.

**Table 3:** Concentrations of As, P, and Se Compounds in *A. officinalis*, Reported as g·P·kg^–1^ (Dry Mass Basis) for Phosphorous and μg·As/Se·kg^–1^ for Arsenic and Selenium^a^

	T-P	E-P	iP	T-Se	E-Se	SeMet 1	SeMet 2	T-As	E-As	DMA	AsV	AsIII
R1	5.2	4.8	4.9	272	149	40	100	18	18	6.6	<3.0	7.3
R2	5.4	6.1	6.0	377	112	16	71	11	14	<3.0	6.0	3.0
R3	6.3	5.8	5.6	246	83	46	120	152	125	84	<3.0	14
R4	6.0	5.0	5.1	87	48	24	76	14	14	<3.0	<3.0	4.4
R5	5.3	5.1	5.1	113	56	28	65	48	26	<3.0	<3.0	24
R6	5.3	5.8	5.7	385	248	106	293	13	14	<3.0	<3.0	7.4
R7	5.9	5.2	5.3	346	212	108	246	34	23	6.8	<3.0	9.0

a Abbreviations: iP, phosphate; T-P, total phosphorus; SeMet, selenomethionine; DMA, dimethylarsinic acid; AsIII, arsenite; and AsV, arsenate. Selenomethionine was measured in the aqueous extract without (SeMet 1) and with (SeMet 2) enzymatic digestion with proteinase K. In the majority of samples, arsenate and DMA were detected, but their concentrations were below the limit of quantification (<3.0 μg·As·kg^1–^).

No report exists on the variability in the elemental metabolome in asparagus samples commercially available to consumers, which is important to study due to significant bioactivity, especially for organosulfur compounds in asparagus.^12^ In this study, examining the sulfur metabolome in asparagus with HPLC-ICP–MS/MS revealed significant variations quantitatively and qualitatively among the studied samples (Supplementary Figure S2). In particular, significant quantitative variation was observed in asparagusic acid, which is a major bioactive sulfur component in asparagus (Table 2) known to display plant growth-inhibiting properties.^13^ The asparagusic acid content varied among the different regions within a wide range (0.02–1.3 g·S·kg^–1^ dry mass basis). This variation could be partly explained by variable oxidation of asparagusic acid to its *S*-oxide forms (Table 2). The origin of this oxidation was investigated by performing extraction under reducing conditions by incorporating 1% w/v ascorbic acid in the extraction solution, ruling out oxidation during sample preparation (Supplementary Figure S3). Interestingly, methionine, another dominant organic organosulfur compound in addition to asparagusic acid, showed a far lower degree of variation among samples than asparagusic acid, with the exception of sample R2, where both methionine and asparagusic acid were significantly lower than in other regions (Table 2). Furthermore, even though methionine and asparagusic acid were comparably dominant species (Table 2) and are both chemically susceptible to S-oxidation, methionine sulfoxide displayed relatively lower concentration in all samples (Figure 2) than asparagusic acid *S*-oxide and a narrower range of variation among samples (0.03–0.08 g·S·kg^–1^). This suggests that, unlike the oxidation of methionine, S-oxidation of asparagusic acid might be enzymatically regulated, which is plausible given that asparagusic acid *S*-oxide also displays plant growth-inhibiting properties similar to those of asparagusic acid,^14^ and therefore, it is plausible that the levels of asparagusic acid and its *S*-oxide would be regulated depending on the growth environment.

**Figure 2:**
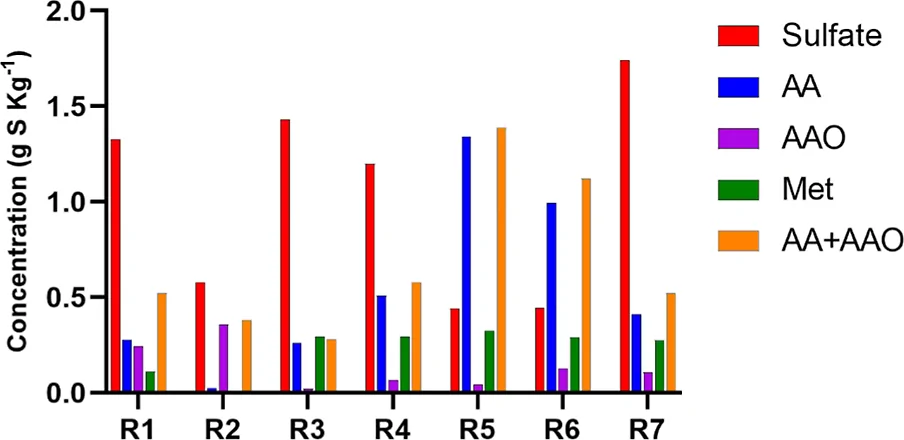
Contrast in the pattern of the major sulfur species detected in *A. officinalis* samples originating in the seven samples of commercially available asparagus labeled to originate from various regions across Europe and South America. The concentrations are reported as g·S·kg^1–^ (dry mass basis). AA, asparagusic acid; AAO, asparagusic acid S-oxide (reported as the sum of the diastereomers); and Met, methionine. AA + AAO, sum of asparagusic acid and its oxide.

The studied asparagus samples represent food products directly available to the consumer. The quantitative and qualitative variations among the different commercially available samples observed in the current study are therefore relevant at various levels, especially in relation to the sulfur profile. First, organosulfur compounds in asparagus have a strong impact on sensory properties of this food as well as on the response of body odors to its consumption, which is commonly reported to be variable among humans due to genetic aspects.^15,16^ Second, organosulfur compounds in asparagus are associated with biological activity, particularly the major compound asparagusic acid, which displays antiparasitic properties^17^ as well as other organosulfur compounds found to display angiotensin-converting enzyme inhibiting function similar to antihypertensive active pharmaceutical ingredients used for managing hypertension.^12^ This renders studying the variability in the sulfur metabolome with HPLC-ICP–MS/MS in asparagus of clear nutritional value.

Several metabolomics studies investigated the composition of green asparagus with a focus on sulfur compounds.^18–20^ Contrary to previous studies, which employed molecular mass spectrometric detection, the present study is the first to investigate the sulfur metabolome in asparagus with element-selective chromatographic detection with ICP–MS. The present study shows that combining ICP–MS detection with molecular mass spectrometric detection is an effective approach for studying the sulfur metabolome in asparagus. This approach enabled nontargeted screening for organosulfur compounds while providing quantitative rather than qualitative information. This was enabled through the much higher resistance of ICP–MS to matrix effects than electrospray ionization-based detection normally used in LC–MS, as well as the ability to quantify sulfur compounds solely based on the elemental response independently of the molecular structure, which is a major advantage of ICP–MS when used for speciation analysis, enabling accurate quantification even in the absence of pure standards.

An additional advantage of ICP–MS detection is the possibility to perform multielemental speciation analysis. As shown in the present work, sulfur, selenium, arsenic, and phosphorus can be measured with ICP–MS/MS, and their elemental profiles can be acquired simultaneously, providing an overview over the distribution of species among these elements and enabling comparison between metalloids and their nonmetal counterparts (i.e., S/Se and As/P). The elemental profiles for arsenic and phosphorus showed higher diversity than that for selenium but far lower than that for sulfur (Figure 1). Inorganic phosphate contributed >95% of the total phosphorus concentration (Table 3), with the appearance of only a few minor unknown phosphorus compounds under the employed chromatographic conditions (Figure 1). On the other hand, at least 5 arsenic compounds were detected (Figure 1). Among the detected compounds, arsenite, arsenate, and DMA were identified by coelution experiments and found to constitute 60%–70% of total arsenic concentration. A major unknown arsenic compound was detected, and its presence was restricted to a single sample labeled (R3), where by far the highest concentrations of total arsenic concentration and DMA were encountered (Figure 1E). Similar to the sulfur metabolome, the arsenic profiles displayed variation among the studied samples. While inorganic arsenic was consistently detected in all samples, its biotransformation product DMA was much more dominant in some samples (e.g., R3) than in others (e.g., R5); see Table 3 for quantitative information and Supplementary Figure S4 for elemental profiles.

Total arsenic concentrations found in asparagus in the present study were generally low with the exception of R3, which showed relatively higher concentrations (Table 3). However, it is well-known that sulfur can replace oxygen in arsenic species, producing thiolated arsenic derivatives that have been reported to display higher toxicity than their nonthiolated counterparts.^21,22^ The fact that asparagus is a plant species that accumulates and biotransforms high concentrations of sulfur adds to the importance of simultaneously monitoring arsenic in this widely consumed food, not only in terms of the total elemental concentration but also through arsenic speciation studies by HPLC–ICP–MS. In the present report, no thiolated arsenic derivative was identified, but this theme has been generally overlooked when characterizing food and is generally applicable to all sulfur-rich edible plants. This is another aspect that highlights the relevance of multielemental speciation analysis with the more recently introduced variation of ICP–MS (tandem ICP–MS detection (ICP–MS/MS)), enabling simultaneous detection of sulfur and arsenic.

## Supplementary Material



## Notes

Figure S1: structural identification with molecular mass spectrometry; Figure S2: variation in sulfur metabolome among studied samples; Figure S3: investigating the dominance of asparagusic acid oxide; and Figure S4: variation in arsenic metabolome profiles in the investigated samples (PDF)
