# Supplementary material for: Characterizing the Elemental Metabolome in Asparagus officinalis with Multielemental Speciation Analysis
Source: J Agric Food Chem. 2026 Aug 28;74(35):28053–9. doi: 10.1021/acs.jafc.6c10753 (PMC13573553; doi:10.1021/acs.jafc.6c10753)

Electronic Supporting Information

Characterizing the elemental metabolome in *Asparagus officinalis* with multi-elemental speciation analysis

Ivan Milovanovic[1], Lorenz Steiner[2], Claudia Kerschbaumer[2], Walter Goessler[2], Bassam Lajin*[2,3]

[1] Innovation Centre of the Faculty of Technology and Metallurgy, University of Belgrade, Karnegijeva 4, 11000 Belgrade, Serbia

[2] Institute of Chemistry, Analytical Chemistry for the Health and Environment, University of Graz, Universitaetsplatz 1, 8010 Graz, Austria

[3] Institute of Chemistry, ChromICP, University of Graz, Universitaetsplatz 1, 8010 Graz, Austria

Corresponding Author: bassam.lajin@uni-graz.at

Contents:

Figure S1: structural identification with molecular mass spectrometry.

Figure S2: variation in sulfur metabolome among studied samples

Figure S3: Investigating the dominance of asparagusic acid oxide.

Figure S4: variation in arsenic metabolome profiles in the investigated samples

Supplementary material Figure S1

Confirmation of the identity of sulfur compounds with molecular mass spectrometry. Multiple mass transitions were monitored including those corresponding to the ^{34}S isotope (M+2) where the sensitivity is sufficient. The graph shows the sulfur metabolome acquired by HPLC-ICPMS/MS at the center and the matching peaks under ESIMS/MS detection. DMSO, dimethylsulfoxide; AAO, asparagusic acid S-oxide (two diastereomers); Met, methionine; MetO, methionine sulfoxide; AA, asparagusic acid.

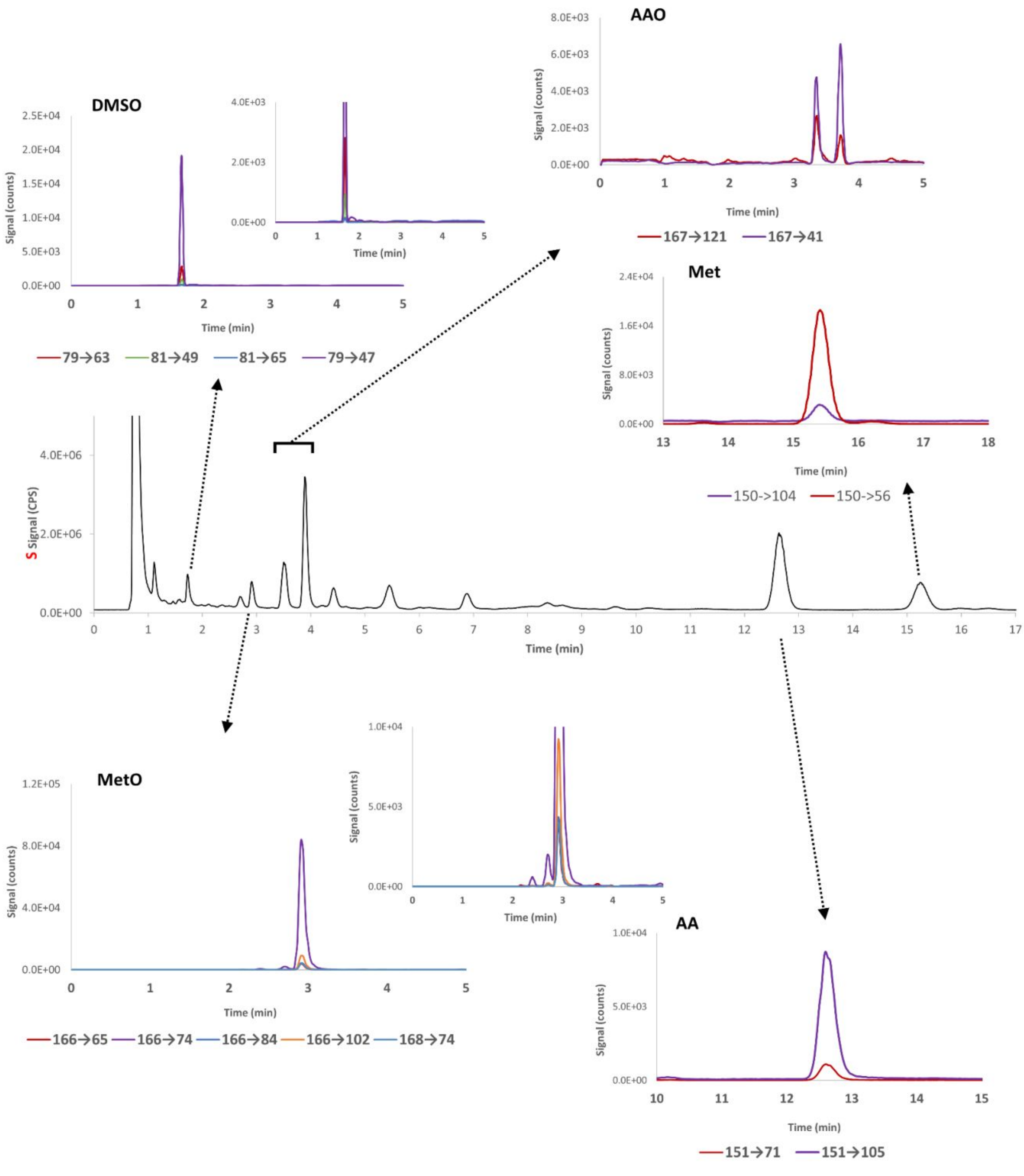

Supplementary material <u>Figure S2</u>

Sulfur metabolomic in A. officinalis from 7 regions across Europe and south America. 1, dimethylsulfoxide; 2, methionine sulfoxide 3, asparagusic acid S-oxide (two diastereomers); 4, asparagusic acid; 5, methionine.

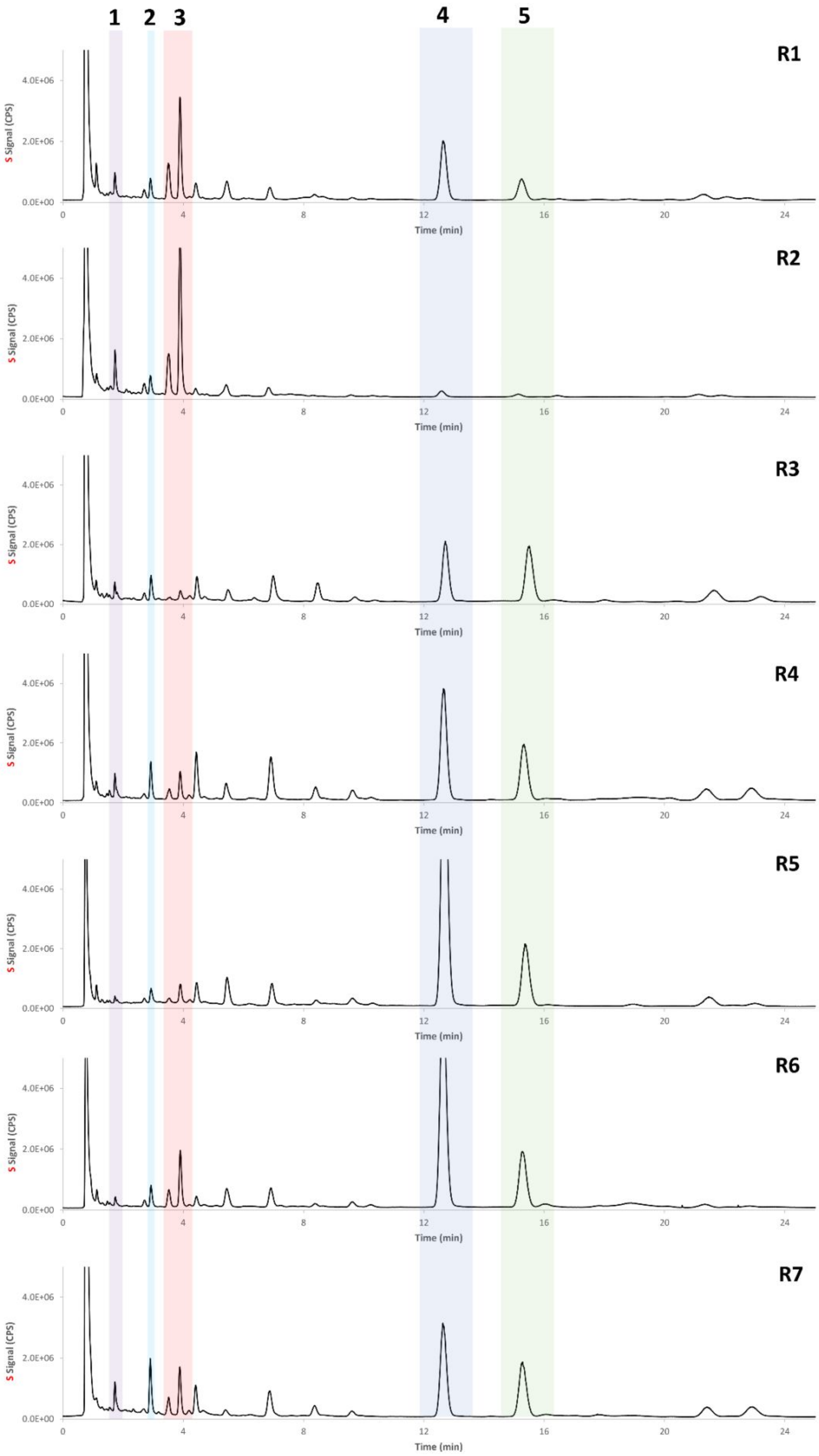

Supplementary material Figure S3

Investigating the dominance of asparagusic acid oxide form (AAO) over asparagusic acid (AA) in sample R2. The chromatograms show the effects of incorporating 1% ascorbic acid in the extraction solution (red) versus pure water (green). The results indicate that most of AAO in R2 does not arise from sample preparation, confirming the observed inter-regional differences.

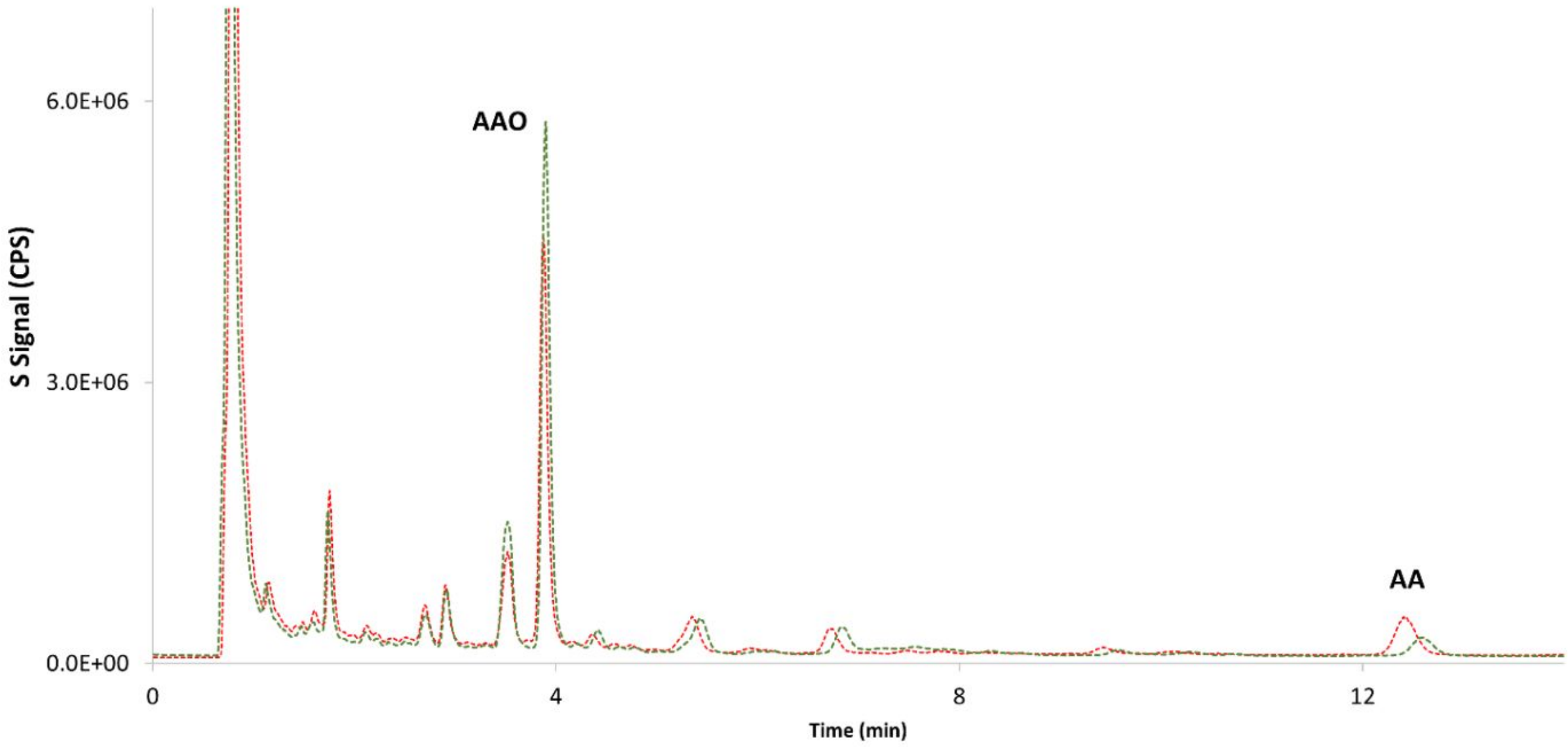

Supplementary material Figure S4

Arsenic metabolomic profiles in A. officinalis from 7 regions across Europe and south America. 1, arsenite; 2, dimethyl arsenic acid; 3, 4 unidentified compounds; 5, arsenate.

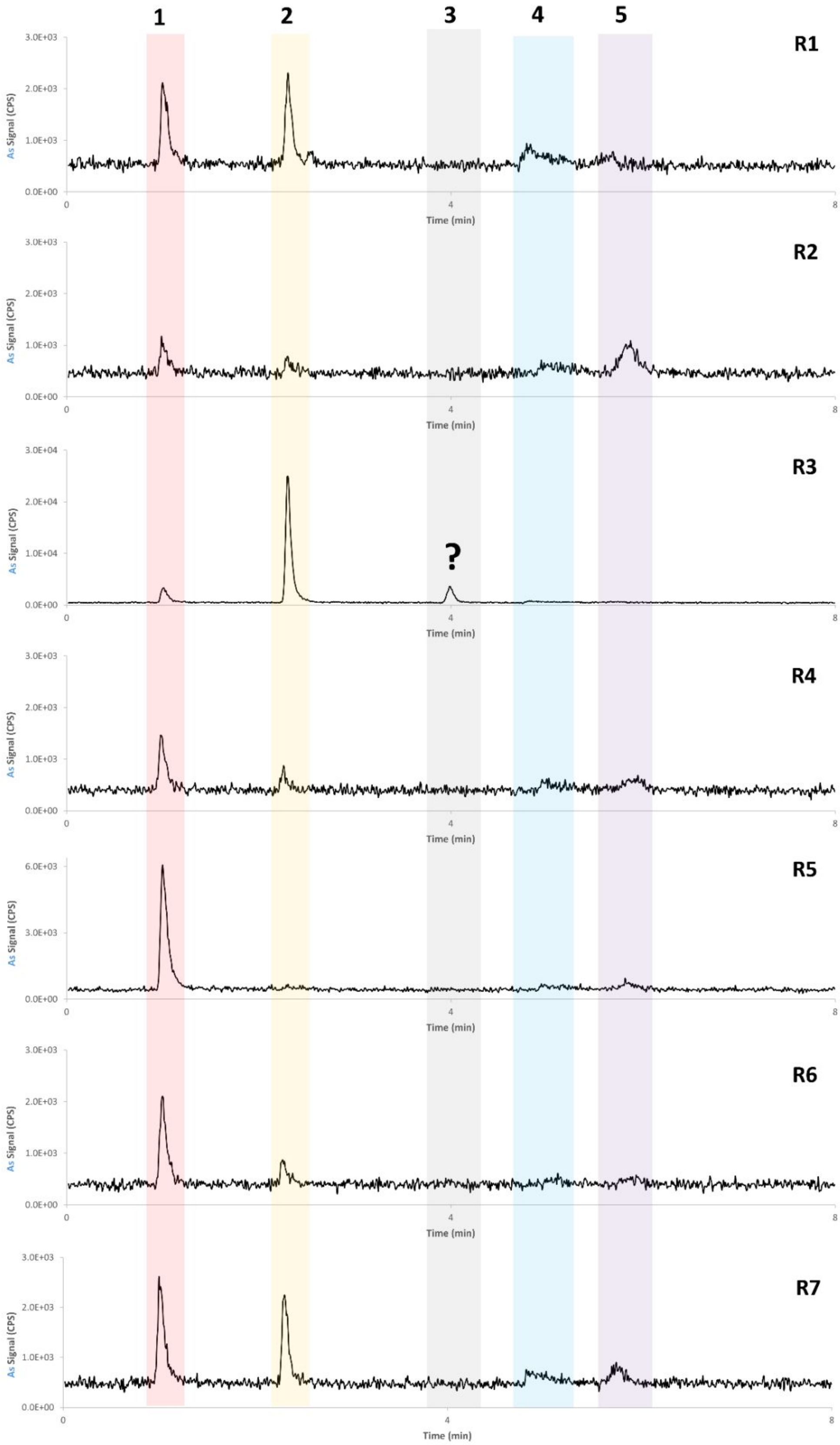

Supplement: Supplementary file 1 [file jf6c10753_si_001.pdf]
